# Coronary steal from an anomalous circumflex artery diagnosed 25 years after ventricular septal defect closure: a case report

**DOI:** 10.1093/ehjcr/ytaf325

**Published:** 2025-07-10

**Authors:** Bastien Provost, Viktoria Weixler, Emmanuelle Fournier, Adelaide Richard, Emre Belli

**Affiliations:** Department of Congenital Heart Diseases, Reference Center for Complex Congenital Cardiac Disease M3C, Marie Lannelongue Hospital, 133 Avenue de la Résistance, Plessis-Robinson 92350, France; Division of Cardiovascular Surgery, The Labatt Family Heart Center, The Hospital of Sick Children, Toronto, ON M5G 1X8, Canada; Division of Cardiovascular Surgery, The Labatt Family Heart Center, The Hospital of Sick Children, Toronto, ON M5G 1X8, Canada; Department of Congenital Heart Diseases, Reference Center for Complex Congenital Cardiac Disease M3C, Marie Lannelongue Hospital, 133 Avenue de la Résistance, Plessis-Robinson 92350, France; Department of Pediatric Cardiology, Hôpital Privé de La Louvière, Lille 59800, France; Department of Congenital Heart Diseases, Reference Center for Complex Congenital Cardiac Disease M3C, Marie Lannelongue Hospital, 133 Avenue de la Résistance, Plessis-Robinson 92350, France

**Keywords:** Case report, Coronary artery anomaly, Congenital heart disease

## Abstract

**Background:**

Association of anomalous origin of a coronary artery arising from pulmonary artery (PA) with other congenital heart defects, such as ventricular septal defects (VSDs), is uncommon. In such cases, coronary anomalies may be overlooked and underdiagnosed.

**Case summary:**

We report a case of late diagnosis of a circumflex coronary artery arising from the right PA, 25 years after VSD-closure during infancy with a complicated post-operative course with severe left ventricular dysfunction. Eventually being discharged, she remained asymptomatic until reaching adulthood, when she developed atypical chest pain during moderate exertion initially with no further investigation being performed. The patient further developed palpitations and underwent a CT-scan, showing an anomalous circumflex artery arising from the right PA. Surgical circumflex artery re-implantation was performed and the symptoms have resolved.

**Discussion:**

This case report highlights the unique pathophysiology of coronary artery steal when arising from the PA. Severe ventricular dysfunction, frequently occurring post-operatively after VSD-closure is usually related to increased afterload. However, other causes should not be neglected especially if function does not improve. Long-term follow-up is therefore mandatory for patients diagnosed with congenital heart disease, even after supposedly simple defects.

Learning points

- Associated lesions should always be tracked, even in case of ‘simple’ congenital heart defects.
- Early referral to a congenital cardiologist for symptoms is mandatory following congenital heart surgery.
- The unique pathophysiology of coronary steal phenomenon can be addressed effectively by coronary re-implantation.

## Introduction

Anomalous origin of the left coronary artery from the pulmonary artery (ALCAPA) is a rare but life-threatening situation, often presenting in early childhood with heart failure. While associated defects like a ventricular septal defect (VSD) are uncommon, they can compensate for the abnormal coronary artery flow. We report a case that illustrates the unique pathophysiology of ALCAPA, and that highlights the importance of long-term follow-up in patients with congenital heart disease, even after supposedly simple lesions and even years after surgical repair.

## Summary figure

**Figure:**
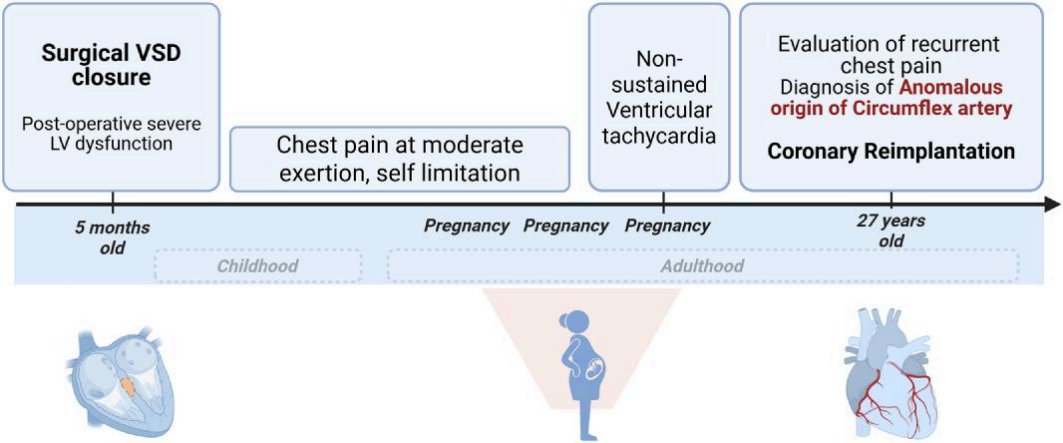


## Case presentation

A 27-year-old woman was referred to our department with a long story of recurrent chest pain. Her medical history included a VSD-closure at 5 months of age. The post-operative course was complicated by severe left ventricular dysfunction, which was attributed to the usual increased afterload post-VSD closure that eventually recovered. Ever since then she has been relatively asymptomatic until adulthood, despite always avoiding physical activity due to long-lasting chest pain. She even successfully went through three pregnancies, even though developing ventricular tachycardia during her third pregnancy, being managed with beta-blockers.

On examination, she was asymptomatic at rest, her clinical status was New York Heart Association II, and her cardiovascular assessment was normal except for a functional systolic murmur on auscultation.

**Figure 1:**
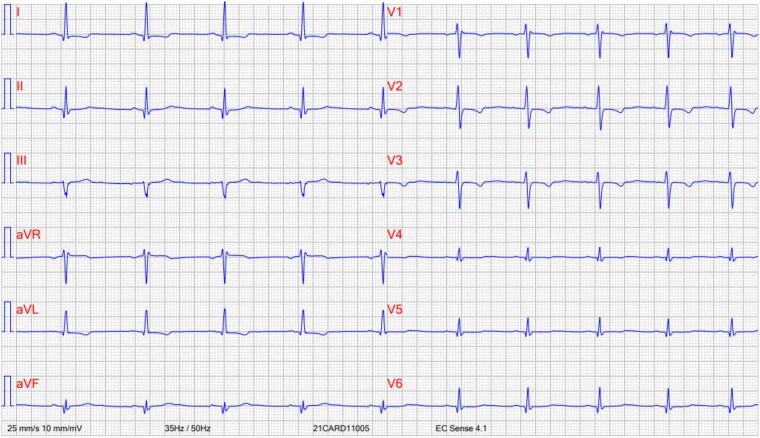
Pre-op ECG showing discrete and a specific signs of ischaemia, and incomplete right bundle branch block.

ECG showed a regular rhythm, incomplete right bundle branch block, an increased amplitude of Q wave in D1, D2, aVL, V5, and V6 as well as T-wave abnormalities in the high and low lateral wall (*Figure 1*).

Transthoracic echocardiography revealed normal intra-cardiac anatomy with an intact VSD patch. Notably, there was a low-velocity ascending flow along the left ventricular lateral wall towards the great arterial trunks (see Supplementary material online, *Video*).

**Figure 2:**
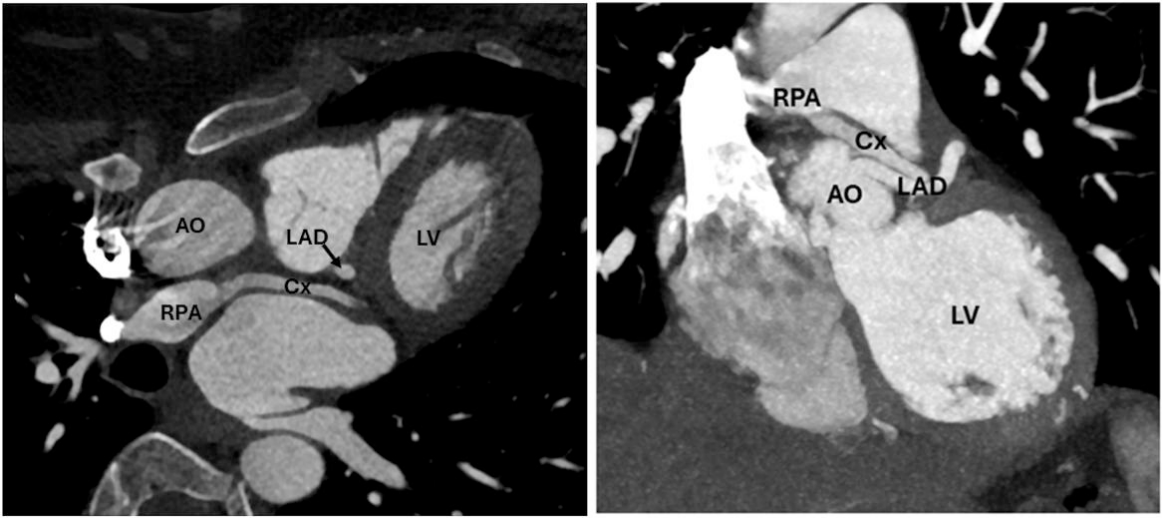
CT-scan showing anomalous arising of the circumflex coronary artery (cx) from the right pulmonary artery (RPA), while the left anterior descending artery (LAD) arises normally form the aorta (AO). LV, left ventricle.

**Figure 3:**
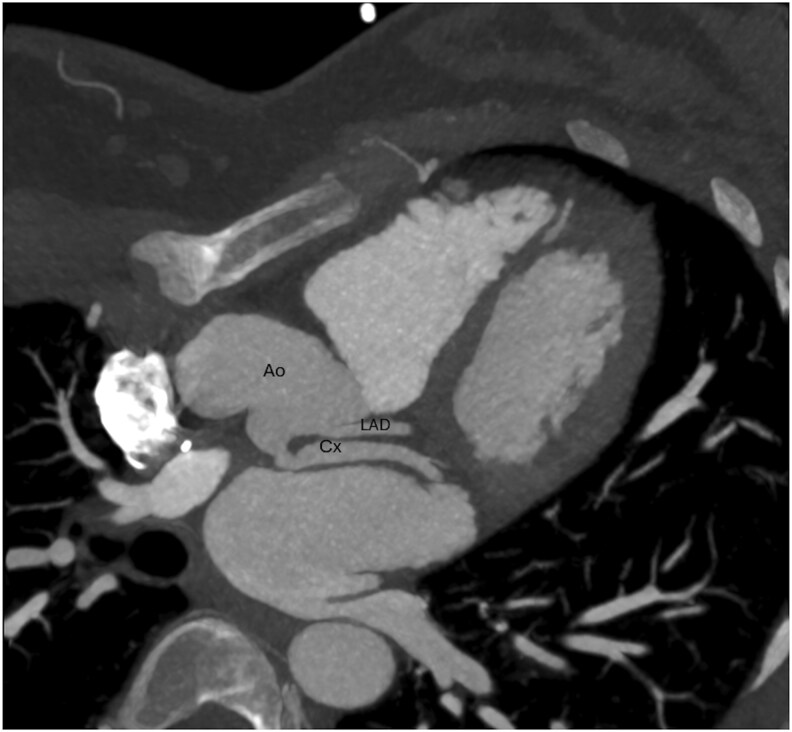
Post-operative CT showing the patent circumflex coronary artery (Cx) after re-implantation into the aorta (Ao) behind the left anterior descending artery (LAD).

Eventually, a computed tomography angiogram showed an anomalous origin of the circumflex coronary artery from the right pulmonary artery (PA), alongside dilation of the left main descending artery and normal right coronary artery anatomy (*Figure 2*). This anomalous connection of the circumflex coronary artery from the PA is a known cause for relative ischaemia of the adjacent myocardium, secondary to the coronary steal phenomenon. In the context of myocardial ischaemia symptoms (previous ventricular tachycardia and typical chest pain at exertion), we preferred not to carry out further tests, and to proceed with surgical repair which consisted of re-implantation of the circumflex coronary artery into the aorta, with the goal to restore antegrade flow. The procedure was performed via midline sternotomy. After dissection of the aortic and pulmonary roots, cardiopulmonary bypass was initiated with an arterial cannula in the ascending aorta and a single venous cannula in the right atrium. The ascending aorta was clamped and transected at the sino-tubular junction, and the main PA was open carefully towards the right PA. The circumflex artery was harvested with a large cuff of PA wall and re-implanted into the ascending aorta. Finally, the defect in the right PA was reconstructed with autologous pericardial patch. Post-operative transoesophageal echocardiogram demonstrated a good result with a good biventricular function, antegrade flow in the transferred circumflex coronary artery and good flow in the main PA and both branch PAs. The patient recovered well and was discharged home on post-operative day 6 with a 3-month course of aspirin. She remains asymptomatic 18 months after the surgery and no longer describes chest pain on moderate exertion. A computed tomography angiogram was performed 6 months after the procedure, confirming an unobstructed circumflex coronary artery from the aorta (*Figure 3*).

## Discussion

**Figure 4:**
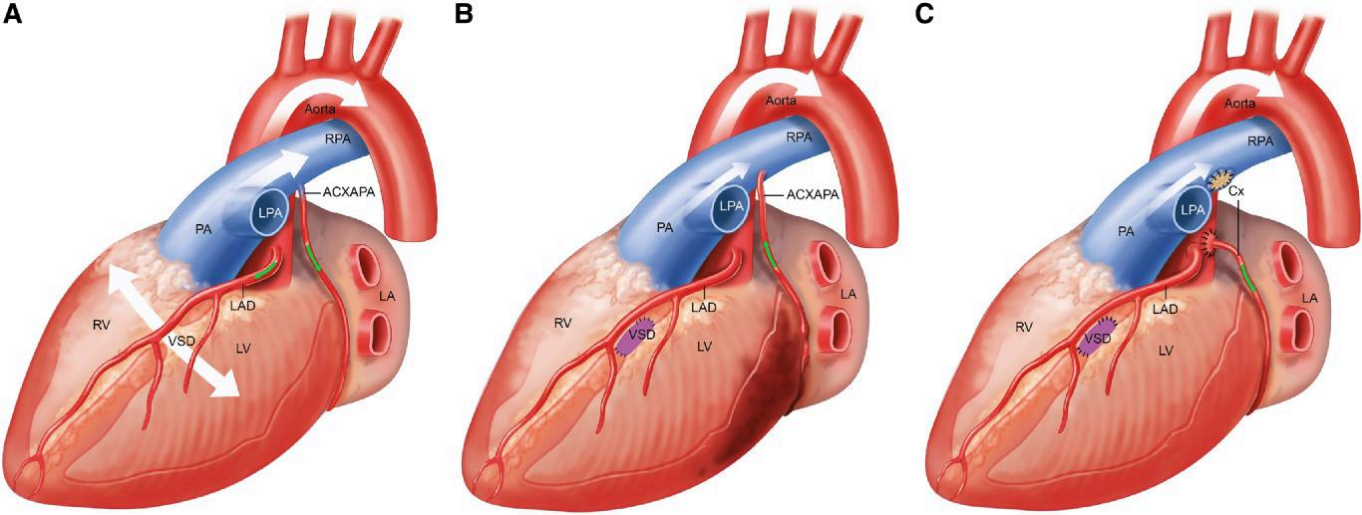
Pathophysiology of the coronary steal before and after ventricular septal defect (VSD) closure. (*A*) Prior to VSD-closure: the shunt through the VSD increased the pulmonary pressure that maintains an antegrade flow in the anomalous circumflex artery (ACXAPA). (*B*) After VSD-closure: the drop of pulmonary pressure leads to a reverse flow in the ACXAPA, thus causing coronary steal and underlying myocardium ischaemia. (*C*) After circumflex coronary artery transfer into the aorta: there is again antegrade flow into the coronary artery without myocardial ischaemia. Thick white arrows indicating high blood flow, thin white arrows indicating low blood flow. Green arrows showing direction of the coronary blood flow.

Anomalous origin of a coronary artery from the PA is typically a life-threatening condition due to coronary steal phenomenon.^1^ Indeed, the flow in the coronary artery is reversed, as it is connected to the low pressure pulmonary arterial system, leading to myocardial ischaemia in the supplied territory. In addition, the left ventricle papillary muscle is often affected by this ischaemia, resulting in a dysfunctional sub-mitral apparatus and mitral valve regurgitation that worsens the left ventricular function and ultimately the prognosis.^2^ To address this coronary steal phenomenon, surgical repair should always be considered. Even though coronary ligation has been described and is theoretically effective from a physiological perspective, coronary re-implantation is the preferred method to restore a two-coronary-artery-anatomy with very good long-term outcomes.^3,4^ In this case, the presence of a large, non-restrictive VSD during infancy masked the coronary anomaly by maintaining elevated pulmonary pressures, thus preventing retrograde flow into the abnormal circumflex coronary artery (*Figure 4A*). After VSD-closure, decreased pulmonary pressures allowed reverse flow through the anomalous circumflex artery, resulting in relative ischaemia, left ventricular dysfunction and subsequent symptoms of chest pain and ventricular tachycardia during exertion (*Figure 4B*). The isolated nature of the circumflex coronary artery’s anomalous connection likely contributed to the patient’s relatively stable clinical course until adulthood,^5,6^ and the symptoms were considered as benign. Eventually, the symptoms resolved after surgical re-implantation of the circumflex coronary artery into the aorta (*Figure 4C*). There are several learning points of this case report for future clinical practice. First of all, a severe left ventricular dysfunction after a straightforward VSD-closure, which is usually caused by the increase of afterload, should always be considered with caution, as it may be caused by an additional associated lesion. Furthermore, this highlights the importance of a close follow-up and patient’s education after correction of congenital heart defects, even in supposedly simple lesions. In our specific case, active investigation should have been considered, especially in the setting of pregnancy surveillance and considering the complicated initial post-operative course. Finally, this case report illustrates the pathophysiology of coronary steal associated with anomalous coronary artery connections.

## Supplementary Material

ytaf325_Supplementary_Data

## Data Availability

The data underlying this article will be shared on reasonable request to the corresponding author.

## Lead author biography

Bastien provost was successively a fellow at the academic Hospital of Brest, France, then at the hospital Marie Lannelongue, France, in cardiac and thoracic surgery, with a keen interest in paediatric pathologies. He then sub-specialized in congenital heart surgery for 6 years at the hospital Marie Lannelongue, one of the leading centres in Europe for Congenital heart diseases, with particular interest in neonatal surgery and valve repair. He is currently a fellow in the Department of Cardiovascular Surgery at the Hospital for Sick Children, Toronto.
